# On the Effects of Virulent Matters When Taken into the Stomachs of Men and the Lower Animals

**Published:** 1852-04

**Authors:** 


*On the Effects of Virulent Matters when taken into the Stomachs of Men and the Lower Animals.*—M. Regnault, director of the Veterinary School at Alfort, gave the following summary of his researches:

1. The dog and pig may eat all the products of secretion, whatever they may be, without prejudice to their health; all the cadaveric debris proceeding from animals suffering under glanders, furuncular disease, rabies, the contagious typhus and pneumonia of oxen, and the contagious epizootics of fowls.

2. It is not the same with fowls, unless perhaps with the exception of their own diseases, in order to determine which it would be necessary to make the experiment out of the epizootic atmosphere, which M. Regnault has not been able to effect.

3. The virulent matters of rabies and acute glanders, which lose their contagious properties by transit through the alimentary canal of the dog or pig, retain them in passing through that of the horse.

4. The matter of the furuncular disease which is taken with impunity by the dog and pig, gives rise to disease when swallowed by the herbivora, the sheep, goat, and horse.

5. Carnivora and omnivora eat the matters with impunity, because their organization is adapted for the conversion and digestion of animal matters, while that of the herbivora is suited only to the digestion of vegetable substances.

6. The flesh of the pig that has eaten these virulent matters is perfectly safe for human food.

7. Culinary operations so change the poisonous nature of the matters proceeding from animals with either of these contagious maladies, that they have been swallowed with impunity by herbivora.

From these conclusions the author draws the inferences, that there is no reason, in a sanitary point of view, for preventing pigs, fowls, &c., being fed on such matters; and that, however repulsive it may seem to the human being, meat, milk, &c., of animals suffering under contagious maladies may be eaten with impunity, if previously cooked or boiled.—*Medical News and Library.*

